# Quantum asymmetry between time and space

**DOI:** 10.1098/rspa.2015.0670

**Published:** 2016-01

**Authors:** Joan A. Vaccaro

**Affiliations:** Centre for Quantum Dynamics, Griffith University, Nathan, Queensland 4111, Australia

**Keywords:** time, violation of time-reversal symmetry, arrow of time

## Abstract

An asymmetry exists between time and space in the sense that physical systems inevitably evolve over time, whereas there is no corresponding ubiquitous translation over space. The asymmetry, which is presumed to be *elemental*, is represented by equations of motion and conservation laws that operate differently over time and space. If, however, the asymmetry was found to be due to deeper causes, this conventional view of time evolution would need reworking. Here we show, using a sum-over-paths formalism, that a violation of time reversal (T) symmetry might be such a cause. If T symmetry is obeyed, then the formalism treats time and space symmetrically such that states of matter are localized both in space and in time. In this case, equations of motion and conservation laws are undefined or inapplicable. However, if T symmetry is violated, then the same sum over paths formalism yields states that are localized in space and distributed without bound over time, creating an asymmetry between time and space. Moreover, the states satisfy an equation of motion (the Schrödinger equation) and conservation laws apply. This suggests that the time–space asymmetry is *not elemental* as currently presumed, and that T violation may have a deep connection with time evolution.

## 1. Introduction

There is nothing unphysical about matter being localized in a region of space; matter can simply exist at one location and not another. But for it to be localized in a finite period of time is altogether different. Indeed, as the matter would exist only for that period and no other, the situation would be a direct violation of mass conservation. In conventional quantum mechanics, this undesirable situation is avoided axiomatically by requiring matter to be represented by a quantum state vector whose norm is fixed over time. There is, however, no corresponding restriction of the state vector over space.

The underlying time–space asymmetry here can be traced to the fact that the state, and the matter it represents, is presumed to undergo continuous translation over time (as time evolution), but there is no corresponding presumption about the state undergoing translations over space. Even in relativistic quantum field theory, where both time and space are treated equally as the coordinates of a space–time background, a similar asymmetry holds, because time evolution and conservation laws are presumed to operate differently over time and space.

Nevertheless, time and space could have an equivalent footing at a fundamental level if any asymmetry between them were to arise *phenomenologically* rather than being imposed axiomatically. Such a prospect is well worth pursuing, because it would help us to understand the relationship between time and space. It would require finding an underlying mechanism that, owing to phenomenological conditions, affects the spatial and temporal translational degrees of freedom in different ways to the extent matter can be localized in space but not in time. This suggests that we should examine the phenomenological character of the operations associated with the translational degrees of freedom. The generators of translations in space and time are given by the momentum and Hamiltonian operators, respectively, and with them lies a difference that sets space and time apart in the quantum regime.

In fact, the last 50 years [1]–[6] have shown that Nature is not invariant to particular combinations of the discrete symmetry operations of charge conjugation (C), parity inversion (P) and time reversal (T). The violation of these discrete symmetries is observed in various particle decays independent of position in space, and so they occur over translations in time and not translations in space. In terms of the corresponding generators, this implies that the Hamiltonian violates the discrete symmetries, whereas the momentum operator does not.

The discrete symmetry violations are accounted for in the standard model of particle physics by the Cabibbo–Kobayashi–Maskawa (CKM) matrix [7],[8]. Studies of the violations have been made in relation to baryogenesis in the early universe [9], the arrow of time and irreversibility [10]–[13], the time operator [14], quantum entanglement and Bell inequalities [15]–[31], decay and decoherence [32],[33], complementarity and quantum information [34]–[36], quantum walks [37] and the potential for T violation to have large-scale physical effects [38],[39]. In particular, in reference [38], I modelled the state of the universe as a superposition of paths that zigzag through time, and showed that T violation can, in principle, affect the time evolution in a global way. Then, in reference [39], I showed that the effect on time evolution is greater when the paths are constructed in the limit of infinitely small steps.

Here we explore the potential impact the violations of the discrete symmetries may have for giving quantum states different representations in space and time. The aim is not to study specific instances of the violations as observed experimentally, but rather to look for possible consequences of the violations in general terms. For this, the definitions of the P and T operations given by Wigner in relation to non-relativistic quantum mechanics [40] are sufficient and so we shall undertake the analysis using the same theory as a basis. Many previous studies have used the same framework [10]–[37]. A relativistic analysis will be left for a future study.

We will need, however, to depart from conventional quantum mechanics in three important ways. The justification for these departures lies in the eventually recovery of the conventional formalism under appropriate conditions. The first departure is that we will not impose any equation of motion, such as the Schrödinger equation, on states because to do so would directly build in the asymmetry between time and space mentioned above. Instead, we anticipate that an effective equation of motion will arise phenomenologically in some way. Second, we will consider states that describe the location of a material object either in space or in time. While the location in space can simply be given by a wave function, say *ψ*(***x***), in the position representation, the location in time is quite unconventional as it would need to be given by a wave function, say *ϕ*(*t*), in what might be called the ‘time representation’. Here, |*ϕ*(*t*)|^2^ gives the probability density for the object being at time *t* just as |*ψ*(***x***)|^2^ gives the probability density for the object being at position ***x***. Note that, in general, *ϕ*(*t*) violates mass conservation as it describes the object as having potential existence at the points in time where |*ϕ*(*t*)|^2^≠0 and definitely not existing at the points where |*ϕ*(*t*)|^2^=0. This lack of mass conservation is the price we must pay to keep the formalism symmetric with respect to time and space. Nevertheless, we anticipate that mass conservation will arise phenomenologically rather than being imposed on the formalism. The third departure from conventional quantum mechanics is that we need to include the P and T symmetry operations in the formalism explicitly, even in the situation where the corresponding symmetries are obeyed and the actions of P and T are redundant. This will ensure that we have a consistent formalism that operates both when the symmetries are obeyed and also when they are violated.

The particular way in which we include the P and T discrete symmetry operators is motivated by the fact that they reverse the directions of spatial and temporal translations, respectively. A one-dimensional path in space that consists of a sequence of translations that alternate in direction can, therefore, be expressed explicitly in terms of parity inversions P. The same can be said for the T operation in relation to a sequence of time translations that alternate in direction. The roles that P and T play will be greater in mathematical constructions that involve a greater number of direction reversals. Such constructions have the potential to display the effects of any discrete symmetry violation to a greater extent. Feynman's path integral formalism immediately comes to mind as one that involves a superposition of all paths that zigzag through configuration space between two states. However, this formalism is inextricably associated with dynamics and thus is tied to the space–time asymmetry mentioned above. We need to develop a different approach if we are to keep time and space on an equal footing at a fundamental level.

Our approach is as follows. We will first consider the effects of P violation on spatial localization. We will begin with a quantum state that represents some material object as being localized in space, and for this, we will need the variance in the object's position to be finite. We will place no other constraint on the position and so we will need a quantum state that yields the least information about position (and thus maximum entropy) for a fixed variance; the optimum pure state fitting this requirement has a Gaussian wave function in the position representation [41]. Note that a classical particle undergoing a one-dimensional Wiener process has a position probability density that is Gaussian; it also has trajectories that consist of infinitely many reversals in direction of the kind we have been considering. With this in mind, we will decompose the Gaussian quantum state into a superposition of infinitely many paths through space where each path has infinitely many reversals in direction. The reversals in the direction of each path will be expressed explicitly in terms of the P symmetry operation and the translations in terms of the momentum operator. We will find that the violation of P symmetry has no effect on the construction. Then, we will apply the same sum-over-paths construction to a quantum state that represents the object as being localized in time, but with the path reversals expressed explicitly in terms of the T symmetry operator and the translations in terms of the Hamiltonian. Using the same construction will ensure that *the formalism is symmetric with respect to the representation of states of matter in both space and time* when the discrete symmetries hold. The situation will be found to change dramatically when T symmetry is violated. Only then will the formalism exhibit a time–space asymmetry that is consistent with conventional quantum mechanics. The important point to be made here is that the asymmetry will not be imposed on the formalism at a fundamental level, but rather it will arise *phenomenologically* due to the T violation.

Given the fundamental character of the issues involved, one should not be surprised to find that to make any progress we need to pay due attention to quite subtle mathematical details. In particular, while the concept of the limit of an infinite sequence has rigorous meaning in a mathematical context, there is no *a priori* reason to suppose that it automatically carries a corresponding value in a theory that is designed to underpin experimental physics. After all, the accuracy of observations made in experimental physics is always restricted by finite resources. For example, consider a theory in which the limit point *a* of the convergent sequence *a*_1_, *a*_2_, *a*_3_, … (i.e. where *a*_*n*_→*a* as n→∞) represents an experimental parameter, and let *ϵ* represent the experimental accuracy of measuring *a* for a given level of resources. The convergence of the sequence implies that there exists a natural number *N*_*ϵ*_ that depends on *ϵ* for which |*a*−*a*_*n*_|<*ϵ* for all *n*>*N*_*ϵ*_, and so it is not possible to physically distinguish (using the given resources), the limit point *a* from any of the terms *a*_*n*_ for *n*>*N*_*ϵ*_. Under such circumstances, the set {*a*_*n*_:*n*>*N*_*ϵ*_} would be more representative of the physical situation than just the limit point *a*. Set representations of this kind will be important for expressing quantum states in a manner which better represents their physical implications.

The structure of the remainder of the paper is as follows. We develop a sum-over-paths construction of a quantum state that is localized in space and examine the effects of the violation of P symmetry in §2. We apply the same construction to quantum states that are localized in time and examine the effects of the violation of T symmetry in §3. Following that, in §4, we show how the conventional Schrödinger equation and conservation of mass emerge as a result of coarse graining over time, and explore how the new formalism might be tested experimentally. We end with a discussion in §5.

## 2. Mathematical construction of quantum states in space

### (a) Developing the construction

We first need to develop the mathematical construction of quantum states that are localized in space and consist of a superposition of infinitely many paths each of which has possibly infinitely many reversals in direction. For this, consider a simple one-dimensional model universe composed of a single ‘galaxy’ as our material object. The galaxy is representative of any spatially localized physical system with mass and could in fact be a star, a planet or just a single particle; its details are not important for this study. The location of the galaxy is described by a set of observables that represent all its spatial degrees of freedom. Imagine that at a particular time, each observable is in some localized state that is uncorrelated with respect to every other observable in the set. This will almost certainly result in the galaxy being far from its minimum energy state, however neither the energetics nor the dynamics are important in this section. In addition, because the same analysis applies to each observable, we will only treat one representative observable explicitly. Let that observable be the centre of mass coordinate, which we assume to have a finite variance. As mentioned above, the best choice for a pure state under these circumstances is one described by a Gaussian wave function [41], which we write as follows

2.1 $$|\psi \rangle \propto \int dx\exp\left(-\frac{x^{2}}{2\sigma_{x}^{2}}\right){\left|x\right\rangle }_{x},$$

where *x* and |*x*〉_*x*_ are the eigenvalue and corresponding eigenstate of $\hat{X}$, the operator representing the *x* component of the centre of mass position, and *σ*_*x*_ is a width parameter. This state can be written explicitly in terms of spatial translations as

**Figure 1.**
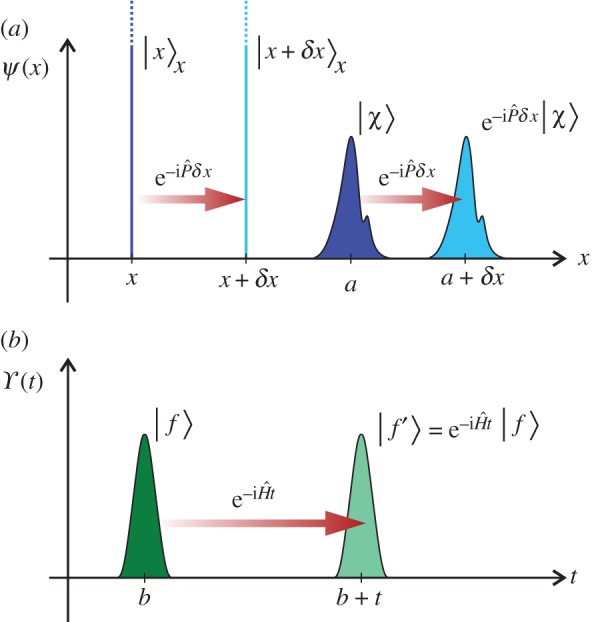
Sketches illustrate the translation of wave functions along (*a*) the *x*-axis and (*b*) the time axis. In (*a*), the wave functions represent the position eigenket |*x*〉_*x*_ and an arbitrary state |*χ*〉 and the translation is by a distance *δx*. In (*b*), the wave function represents the state |*f*〉 and the translation is by an interval *t*.

2.2 $$|\psi \rangle \propto \int dx\exp\left(-\frac{x^{2}}{2\sigma_{x}^{2}}\right)\exp(-i\hat{P}x){\left|0\right\rangle }_{x},$$

where the operator representing the total momentum of the galaxy, $\hat{P}$, generates spatial translations according to

$$\exp(-i\hat{P}\delta x){\left|x\right\rangle }_{x}={\left|x+\delta x\right\rangle }_{x}$$

as illustrated in figure 1*a*. Here and throughout this paper, we use units in which ℏ=1. Inserting the resolution of the identity $\hat{1}=\int dp{\left|p\right\rangle }_{p}{\;}_{p}\langle p|$ into equation (2.2) gives

$$|\psi \rangle \propto \iint dx\;dp\exp\left(-\frac{x^{2}}{2\sigma_{x}^{2}}\right)\exp(-ipx){\left|p\right\rangle }_{p}{\;}_{p}{\left\langle p|0\right\rangle }_{x},$$

where $\left\{{\left|p\right\rangle }_{p}:\hat{P}{\left|p\right\rangle }_{p}=p{\left|p\right\rangle }_{p}\right\}$ is the momentum basis. Carrying out the Fourier transform with respect to *x* yields $|\psi \rangle \propto \exp(-\frac{1}{2}{\hat{P}}^{2}\sigma_{x}^{2}){\left|0\right\rangle }_{x}$ and making use of the result

2.3 $$\exp\left(-\frac{A^{2}}{2}\right)=\lim_{N\to \infty }\cos^{N}\left(\frac{A}{\sqrt{N}}\right)$$

then leads to

2.4 $$|\psi \rangle \propto \lim_{N\to \infty }\frac{1}{2^{N}}{\left[\exp\left(i\frac{\hat{P}\sigma_{x}}{\sqrt{N}}\right)+\exp\left(-i\frac{\hat{P}\sigma_{x}}{\sqrt{N}}\right)\right]}^{N}{\left|0\right\rangle }_{x}.$$

Expanding the *N*-fold product in equation (2.4) gives a series of terms each of which comprise *N* translations (or ‘steps’) of $\pm \sigma_{x}/\sqrt{N}$ along the *x*-axis. For example, a term of the form

**Figure 2.**
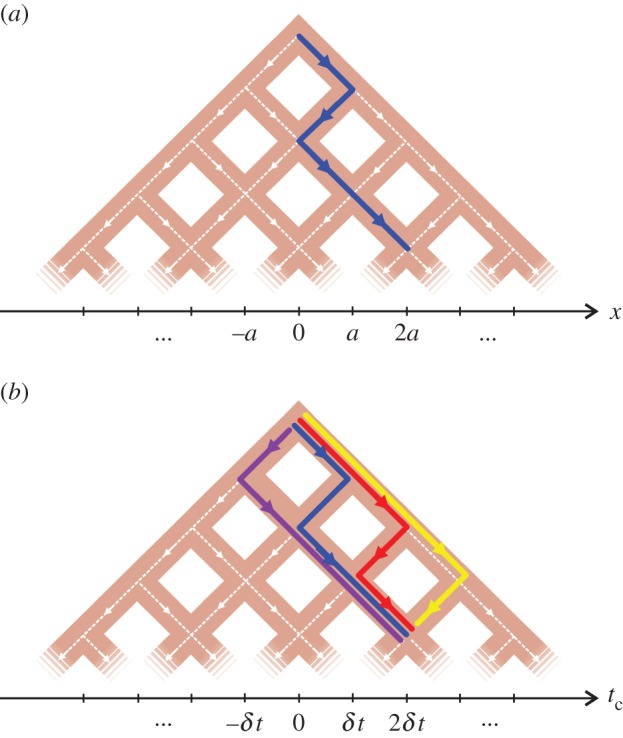
Binary tree diagrams represent virtual paths in (*a*) space and (*b*) time. Each edge (white dashed line) in the tree represents a virtual displacement along the black horizontal axis. The thick blue edges in (*a*) represents a virtual path that passes through the sequence of points 0, *a*, 0, *a*, 2*a* on the *x*-axis. In (*b*), four different virtual paths from 0 to 2*δt* on the *t*_*c*_ axis are represented in the tree by thick edges coloured yellow, red, blue and purple.

2.5 $$\cdots \exp\left(-i\hat{P}a\right)\exp\left(-i\hat{P}a\right)\exp\left(i\hat{P}a\right)\exp\left(-i\hat{P}a\right){\left|0\right\rangle }_{x},$$

where $a=\sigma_{x}/\sqrt{N}$, describes a path on the *x*-axis from the origin 0 through the sequence of points *a*, 0, *a*, 2*a* and so on, as illustrated in figure 2*a*. Equation (2.4) can be viewed, therefore, as a superposition of random paths away from the origin |0〉_*x*_ in the limit of infinitely small steps, and shares similarities with both quantum walks [42] and Feynman's sum over paths [43]. Note that here, however, the random path is traversed without reference to time, and so it should be considered to be traversed in a zero time interval. Each random path is, therefore, a generalization of the virtual displacements in D’Alembert's principle in classical mechanics [44]. For this reason, each individual path shall be called a *random virtual path* and the superposition of a set of random virtual paths like that in equation (2.4) shall be called a *quantum virtual path*.

Although they share similarities, a quantum virtual path is quite distinct from Feynman's sum over paths [43]. For example, Feynman's method is used to calculate the probability amplitude for a system to evolve from one state to another. The paths represent potential classical trajectories between the same starting and ending points and the sum gives the total probability amplitude for evolving between the points. In contrast, a quantum virtual path represents a single state. The accumulated displacement over one random virtual path, like that in equation (2.5), gives a potential classical position of the system, and the whole quantum virtual path represents the state given by equation (2.4) in terms of a distribution of potential classical positions. Moreover, calculating the inner product of two states where one (or both) is represented by a quantum virtual path would result in a Feynman-like sum over paths calculation. So in this sense, a quantum virtual path is a *precursor* of Feynman's sum over paths.

The right-hand side of equation (2.4) is not the only way to decompose the state in equation (2.1). But what makes equation (2.4) special is that it consists of a superposition of an infinite number of continuous paths with the property that if one path is picked at random, it will effectively consist of a sequence of infinitesimal segments where each segment has an equal likelihood of representing a step in the positive or negative *x* directions. The set of paths is unbiased with respect to direction in this sense. Another feature that sets the decomposition in equation (2.4) apart is that it comprises all possible paths. The justification of why it should is that in decomposing the state in equation (2.1) in terms of a superposition of paths, we have no reason for leaving out particular paths or, alternatively, for including only particular paths; in the absence of such reasons, all possible paths should be included.

As N→∞, the step length $\sigma_{x}/\sqrt{N}$ in equation (2.4) will eventually breach the fundamental lower bound, say *δx*_min_, that is expected for physically distinguishable positions. For example, there are reasons [45] to believe that points in space are indistinguishable at the scale of the Planck length ℓ_*P*_≈1.6×10^−35^ m. Let *N*^(space)^_min_ be the value of *N*, where the step length $\sigma_{x}/\sqrt{N}$ becomes equal to *δx*_min_, i.e. $N_{\min}^{\left(\mathrm{space}\right)}=\sigma_{x}^{2}/\delta x_{\min}^{2}$. This implies that the limit on the right-hand side of equation (2.4) can be replaced by a term corresponding to any value of *N* larger than $N_{\min}^{\left(\mathrm{space}\right)}$ without any physically meaningful consequences. There are an infinite number of such terms, each of which has an *equal status* in representing the state of the universe. They form the set

2.6 $$\psi =\left\{{\left|\psi \right\rangle }_{N}:N\geq N_{\min}^{\left(\mathrm{space}\right)}\right\},$$

where

2.7 $${\left|\psi \right\rangle }_{N}=\frac{1}{2^{N}}{\left[{\hat{P}}^{-1}\exp\left(-i\frac{\hat{P}\sigma_{x}}{\sqrt{N}}\right)\hat{P}+\exp\left(-i\frac{\hat{P}\sigma_{x}}{\sqrt{N}}\right)\right]}^{N}{\left|0\right\rangle }_{x}.$$

In equation (2.7), we have written the translations explicitly in terms of the parity inversion operator $\hat{P}$. It has the property that

2.8 $$\exp\left(i\hat{P}x^{\prime }\right)={\hat{P}}^{-1}\exp\left(-i\hat{P}x^{\prime }\right)\hat{P}$$

which expresses the fact that a translation along the *x* axis by −*x*′ (left side of equation (2.8)) can be produced by first performing a parity inversion, translating by *x*′ and then reversing the parity inversion (right-hand side). Every element in the set ***ψ*** can serve equally well as a representation of the state in equation (2.1) as far as the physically distinguishable spatial limit allows; they all have equal status in this respect.

The mathematical construction represented by equations (2.6) and (2.7) is in the form of the explicit translations and discrete symmetry operations that we need for comparing the difference between quantum states in space and time. Although being equivalent to equation (2.1), we shall henceforth regard equations (2.6) and (2.7) as being a *more fundamental* description of the state of the galaxy owing to this explicit form. Note that the interpretation of equation (2.7) in terms of quantum virtual paths does not hinge on the state |0〉_*x*_ being the eigenstate of position with zero eigenvalue. In fact, any state |*χ*〉 with a variance in position very much smaller than $\sigma_{x}^{2}/2$ (and, correspondingly, a variance in total momentum very much larger than $1/2\sigma_{x}^{2}$) could be used in its place; in that case, the steps in a path represent translations of |*χ*〉 along the *x* axis, as illustrated in figure 1*a*, rather than steps along the *x* axis itself. While this situation allows some ambiguity in the formalism, it does not have any effect on results provided that corresponding adjustments to |*ψ*〉 and *σ*_*x*_ are duly taken.

### (b) Parity inversion invariance and its violation

Given that the Hamiltonian does not appear explicitly in the construction of spatial states, we should not expect to find any effects of discrete symmetry violation here. In particular, *regardless of whether the galaxy obeys parity inversion symmetry or not*

2.9 $${\hat{P}}^{-1}\hat{P}\;\hat{P}=-\hat{P}$$

always holds, and so equation (2.7) can be rewritten as a binomially weighted superposition of spatially translated states, i.e.

2.10 $${\left|\psi \right\rangle }_{N}=\sum_{n=0}^{N}B_{n}\exp\left[-i(2n-N)\frac{\hat{P}\sigma_{x}}{\sqrt{N}}\right]{\left|0\right\rangle }_{x},$$

where

2.11 $$B_{n}=\frac{1}{2^{N}}\left(\begin{array}{c} N\\ n \end{array}\right).$$

In the large *N* limit, |*ψ*〉_*N*_ tends to the Gaussian state |*ψ*〉 in equation (2.1), i.e.

2.12 $$\lim_{N\to \infty }{\left|\psi \right\rangle }_{N}\propto \int dx\;g(x,\sigma_{x}){\left|x\right\rangle }_{x},$$

where

2.13 $$g(x,\sigma_{x})=\exp\left(-\frac{x^{2}}{2\sigma_{x}^{2}}\right).$$

**Figure 3.**
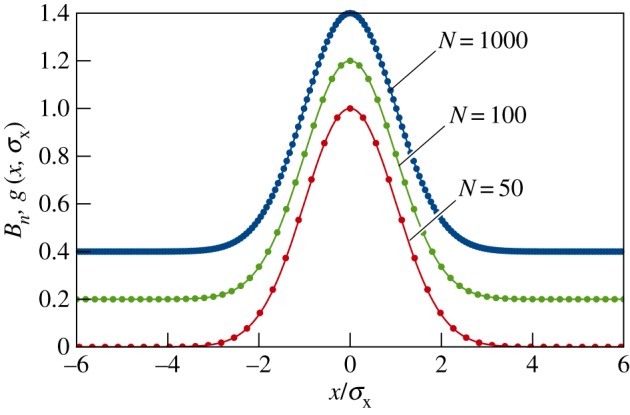
The position representation of the state |*ψ*〉_*N*_ as a function of the scaled position *x*/*σ*_*x*_. The dots represent the exact values *B*_*n*_ given by equation (2.11), and the solid curves represent the Gaussian approximation *g*(*x*,*σ*_*x*_) given by equation (2.13). The abscissae for the discrete coefficients *B*_*n*_ are given by $x/\sigma_{x}=(2n-N)/\sqrt{N}$ in accord with equation (2.10). For clarity, *B*_*n*_ and *g*(*x*,*σ*_*x*_) have been scaled to give a maximum of unity, and the green (*N*=100) and blue (*N*=1000) dots and curves have been displaced vertically by 0.2 and 0.4, respectively.

Figure 3 compares the coefficients *B*_*n*_ (shown as dots) with their large-*N* limit *g*(*x*,*σ*_*x*_) (continuous curves) for a number of different *N* values. The values of *N* have been chosen purposely to exaggerate the discreteness of the state |*ψ*〉_*N*_ in comparison with the limiting state |*ψ*〉 from equation (2.1). In truth, for every |*ψ*〉_*N*_∈***ψ*** in equation (2.6), the values of *N* are sufficiently large (viz. *N*≥*N*^(space)^_min_) that the dots representing *B*_*n*_ for consecutive *n* values are physically indistinguishable, and the locus of points representing *B*_*n*_ is essentially equivalent to the curve *g*(*x*,*σ*_*x*_) up to a proportionality constant. As a consequence, every |*ψ*〉_*N*_∈***ψ*** is physically indistinguishable from the state |*ψ*〉 in equation (2.1).

## 3. Applying the construction to quantum states in time

### (a) Adapting the construction

We now use our construction to explore the temporal analogy of equation (2.1) in which the galaxy is represented in time rather than space. We begin by recalling that the Hamiltonian $\hat{H}$ generates translations through time according to

$$\exp\left(-i\hat{H}t\right)\left|\;f\right\rangle =\left|\;f^{\prime }\right\rangle ,$$

where | *f*〉 and |*f*′〉 represent states at times differing by *t*, as illustrated in figure 1*b*. Next, we construct a set of states analogous to equation (2.6) but with each state representing a superposition of random virtual paths through *time* as

3.1 $$\upsilon_{\lambda }=\left\{{\left|\Upsilon_{\lambda }\right\rangle }_{N}:N\geq N_{\min}^{\left(\mathrm{time}\right)}\right\},$$

where

3.2 $${\left|\Upsilon_{\lambda }\right\rangle }_{N}\propto \frac{1}{2^{N}}{\left[{\hat{T}}^{-1}\exp\left(-i\frac{\hat{H}\sigma_{t}}{\sqrt{N}}\right)\hat{T}+\exp\left(-i\frac{\hat{H}\sigma_{t}}{\sqrt{N}}\right)\right]}^{N}|\phi \rangle .$$

Here λ distinguishes different physical situations that will be specified later, $N_{\min}^{\left(\mathrm{time}\right)}=\sigma_{t}^{2}/\delta t_{\min}^{2}$ is the value of *N* for which the step size $\sigma_{t}/\sqrt{N}$ reaches some fundamental resolution limit in time *δt*_min_ (e.g. taking the resolution limit as the Planck time would mean that *δt*_min_=5.4×10^−44^ *s*), and $\hat{T}$ is Wigner's time reversal operator [40]. The state |*ϕ*〉 plays the role of |0〉_*x*_ in equation (2.7) and is assumed to be sharply defined in time and, correspondingly, to have a broad distribution in energy [46]. More specifically, |*ϕ*〉 must have a variance in energy that is very much larger than 1/2*σ*^2^_*t*_ in analogy with the requirement for any state |*χ*〉 to be used in place of |0〉_*x*_. Other details of |*ϕ*〉 are not crucial for our main results.

It is perhaps worth elaborating a little on what is meant by |*ϕ*〉 being sharply defined in time given that there are well-known difficulties associated with defining an operator to represent time [46],[47]. Fortunately, the absence of a universally accepted time operator does not prevent uncertainties in time from being physically meaningful. Rather, we can use the fact that the Hamiltonian is the generator of translations in time to probe the time uncertainty of a state. For example, $\left|\varphi_{\tau }\rangle =\exp(-i\hat{H}\tau )|\varphi_{0}\right\rangle$ represents the state |*φ*_0_〉 translated in time by *τ*. If the overlap 〈*φ*_*τ*_|*φ*_0_〉 is negligible for all values of *τ* except for |*τ*|≈0, then |*φ*_0_〉 can be regarded as sharply defined in time, at least for the purposes needed here. A more rigorous definition of such states is given by Moyer's timeline states [47]. The ambiguity mentioned at the end of §2*a* also occurs here regarding the choice of the state |*ϕ*〉, and can be treated in a similar way. We will return to this point in §4a.

The violation of T symmetry is expressed by ${\hat{T}}^{-1}\hat{H}\hat{T}\neq \hat{H}$ which implies that there are two versions of the Hamiltonian [38],[39]. We label the two versions as ${\hat{H}}_{F}=\hat{H}$ and ${\hat{H}}_{B}={\hat{T}}^{-1}\hat{H}\hat{T}$. In this construction, one direction of time is not physically distinguishable from the other and the subscripted labels *F* and *B* simply refer to opposite directions; nevertheless, it may be convenient to think of the labels as referring to the customary ‘forwards’ and ‘backwards’ directions of time. Using these definitions together with the fact [40] that ${\hat{T}}^{-1}i\hat{T}=-i$ then gives

3.3 $${\left|\Upsilon_{\lambda }\right\rangle }_{N}\propto \frac{1}{2^{N}}{\left[\exp\left(i{\hat{H}}_{B}\delta t\right)+\exp\left(-i{\hat{H}}_{F}\delta t\right)\right]}^{N}|\phi \rangle ,$$

where, for convenience, we have set

3.4 $$\delta t=\frac{\sigma_{t}}{\sqrt{N}}$$

as the step in time.

Equation (3.3) shows that ${\hat{H}}_{F}$ and ${\hat{H}}_{B}$ are responsible for translations in opposite directions of time. This is an important point that warrants particular emphasis: a translation in time in the opposite direction to that given by $\exp(-i{\hat{H}}_{F}t)$ is not produced by its *inverse* $\exp(i{\hat{H}}_{F}t)$ but rather by its *time reverse*:

$$\exp\left(i{\hat{H}}_{B}t\right)={\hat{T}}^{-1}\exp\left(-i{\hat{H}}_{F}t\right)\hat{T}.$$

Evidently, we need to associate the operators $\exp(-i{\hat{H}}_{F}t)$ and $\exp(i{\hat{H}}_{B}t)$ with *physical evolution* in different directions of time according to equation (3.3). This leaves their respective inverses $\exp(i{\hat{H}}_{F}t)$ and $\exp(-i{\hat{H}}_{B}t)$ to be associated with the *mathematical operations of rewinding* that physical evolution. In short, physical time evolution is described by the former pair of operators, and not by the latter.

In fact, these associated meanings follow from conventional quantum mechanics. For example, let | *f*(*t*)〉 represent the state of an arbitrary closed system at time *t*. Unitary evolution implies that

3.5 $$|\;f(t)\rangle =\exp\left(-i\hat{h}t\right)|\;f(0)\rangle ,$$

where | *f*(0)〉 is the state at *t*=0 and $\hat{h}$ is the corresponding Hamiltonian. Recall that Wigner's time-reversal operator $\hat{T}$ reverses the direction of all momenta and spin [40]. Let the time-reversed states at times 0 and *t* be $\left|b(0)\rangle ={\hat{T}}^{-1}|\;f(0)\right\rangle$ and $\left|b(-t)\rangle ={\hat{T}}^{-1}|\;f(t)\right\rangle$, respectively. Using $\hat{T}{\hat{T}}^{-1}=\hat{1}$ and rearranging shows that $\left|b(-t)\rangle =\exp(i{\hat{T}}^{-1}\hat{h}\hat{T}t){\hat{T}}^{-1}|f(0)\right\rangle$, i.e.

3.6 $$|b(-t)\rangle =\exp\left(i{\hat{T}}^{-1}\hat{h}\hat{T}t\right)|b(0)\rangle ,$$

and so the time-reversed state |*b*(−*t*)〉 represents the evolution from the time-reversed state |*b*(0)〉 according to the Hamiltonian ${\hat{T}}^{-1}\hat{h}\hat{T}$ for the time −*t*. That is, evolving from the state |*f*(0)〉 for the time *t* with the Hamiltonian $\hat{h}$ is equivalent to evolving from the time-reversed state |*b*(0)〉 for the time −*t* with the Hamiltonian ${\hat{T}}^{-1}\hat{h}\hat{T}$. Clearly, $\hat{h}$ generates translations in one direction of time and ${\hat{T}}^{-1}\hat{h}\hat{T}$ generates translations in the opposite direction, which is consistent with equation (3.3).

If our model universe satisfied T symmetry, ${\hat{H}}_{F}$ and ${\hat{H}}_{B}$ would be commuting operators and the terms in equation (3.3) would be able to be manipulated algebraically in exactly the same way as those in equation (2.7) were manipulated to give equation (2.10). Thus, for the temporal quantum virtual path to be qualitatively distinct from the spatial one, the model universe must violate T symmetry to the extent of giving a non-zero commutator $\left[{\hat{H}}_{F},{\hat{H}}_{B}\right]$. We could model such a commutator using details of the T violation that has been observed in the decay of mesons [3]–[6] or that has been speculated for a Higgs field [48],[49]. However, the potential repercussions of T violation will be manifest most clearly for the simplest departure from time-reversal invariance. Accordingly, we shall imagine that our model universe contains an unspecified T-violating mechanism that is consistent with the commutator

3.7 $$\left[{\hat{H}}_{B},{\hat{H}}_{F}\right]=i\lambda$$

for real-valued λ. This is the origin of the parameter λ that appears in equations (3.1) and (3.2).

Equations (6) and (8) of reference [38] show that the operator on the right-hand side of equation (3.3) can be expanded and reordered using the Zassenhaus formula [50] as follows

3.8 $$\begin{array}{rl} {\left[\exp(i{\hat{H}}_{B}\delta t)+\exp(-i{\hat{H}}_{F}\delta t)\right]}^{N} & =\sum_{n=0}^{N}\exp\left[i{\hat{H}}_{B}(N-n)\delta t\right]\exp(-i{\hat{H}}_{F}n\delta t)\\ & \;\times \sum_{v=0}^{N-n}\cdots \sum_{\ell =0}^{s}\sum_{k=0}^{\ell }\exp\left[(v+\cdots +\ell +k)(\delta t^{2}[{\hat{H}}_{F},{\hat{H}}_{B}]+\hat{Q})\right], \end{array}$$

where $\hat{Q}$ contains terms representing higher-order commutators of the form $\left[[{\hat{H}}_{B},{\hat{H}}_{F}],\ldots \right]$. It follows from equation (3.7) that $\hat{Q}=0$ here. Substituting equation (3.8) into equation (3.3) and then simplifying the resulting expression using equations (B.14) and (B.15) in appendix B of reference [38] yields

3.9 $${\left|\Upsilon_{\lambda }\right\rangle }_{N}\propto \sum_{n=0}^{N}I_{N-n,n}(\delta t^{2}\lambda )\exp[i{\hat{H}}_{B}(N-n)\delta t]\exp[-i{\hat{H}}_{F}n\delta t]|\phi \rangle ,$$

where

3.10 $$I_{N-n,n}(z)=\exp\left[-\frac{in(N-n)z}{2}\right]\prod_{q=1}^{n}\frac{\sin[(N+1-q)z/2]}{\sin(qz/2)}$$

is an *interference function* that takes account of the non-commutativity of ${\hat{H}}_{F}$ and ${\hat{H}}_{B}$.

To relate this to what an observer in the galaxy would see, imagine that the galaxy contains a clock that is constructed from T-invariant matter. We will refer to any time shown by the clock as ‘clock time’ and use the symbol *t*_*c*_ to represent its value. Let the state |*ϕ*〉 represents the clock showing the time *t*_*c*_=0. The state

3.11 $$\exp[i{\hat{H}}_{B}(N-n)\delta t]\exp[-i{\hat{H}}_{F}n\delta t]|\phi \rangle$$

represents evolution by $\exp[-i{\hat{H}}_{F}n\delta t]$ in one direction of time followed by $\exp[i{\hat{H}}_{B}(N-n)\delta t]$ in the opposite direction which, by convention, first increases *t*_*c*_ by *nδt* and then decreases it by (*N*−*n*)*δt*, respectively. The state in equation (3.11) would therefore represent the clock showing the net clock time of

3.12 $$t_{c}=(2n-N)\delta t,$$

and so the state in equation (3.9) represents a weighted superposition of states over the range of net clock times from *t*_c_=−*Nδt* to *Nδt*.

### (b) Time-reversal invariance

It is useful to first consider the special case where the universe is invariant under time reversal. For this, we set λ=0, ${\hat{H}}_{F}={\hat{H}}_{B}=\hat{H}$ in equation (3.9). The interference function for λ=0 is the binomial coefficient $I_{N-n,n}(0)=({\;}_{n}^{N})$ and so

3.13 $${\left|\Upsilon_{0}\right\rangle }_{N}\propto \sum_{n=0}^{N}B_{n}\exp[-i(2n-N)\hat{H}\delta t]|\phi \rangle ,$$

where *B*_*n*_ is given by equation (2.11). The coefficient *B*_*n*_ becomes proportional to the Gaussian function $\exp[-{\left(2n-N\right)}^{2}/2N]$ for large *N* and so

$${\left|\Upsilon_{0}\right\rangle }_{N}\underset{∼}{\propto }\sum_{n=0}^{N}\exp\left[-\frac{{\left(2n-N\right)}^{2}}{2N}\right]\exp[-i(2n-N)\hat{H}\delta t]|\phi \rangle .$$

Re-expressing the summation in terms of the index *m*=2*n*−*N* and using the definition $\delta t=\sigma_{t}/\sqrt{N}$ then yields

3.14 $${\left|\Upsilon_{0}\right\rangle }_{N}\underset{∼}{\propto }\sum_{m\in S}\exp\left[-\frac{{\left(m\delta t\right)}^{2}}{2\sigma_{t}^{2}}\right]\exp(-i\hat{H}m\delta t)|\phi \rangle ,$$

where *S*={−*N*,−*N*+2,…,*N*}. We define the large-*N* limit as

3.15 $$|\Upsilon_{0}\rangle =\lim_{N\to \infty }{\left|\Upsilon_{0}\right\rangle }_{N}\propto \int dt\;g(t,\sigma_{t})\exp(-i\hat{H}t)|\phi \rangle ,$$

where *g*(*t*,*σ*_*t*_) is given by equation (2.13). Although figure 3 is explicitly for the spatial case, it can also be used here as a comparison of *B*_*n*_ and *g*(*t*,*σ*_*t*_) in equations (3.13) and (3.15) provided we interpret the horizontal axis as *t*/*σ*_*t*_. Likewise, for *N*≥*N*^(time)^_min_, the locus of points representing *B*_*n*_ is essentially equivalent to the curve *g*(*t*,*σ*_*t*_) up to a proportionality constant, and so every |*Υ*_0_〉_*N*_∈***υ***_0_ in equation (3.1) is physically indistinguishable from the state |*Υ*_0_〉 in equation (3.15).

Hence, for time-reversal invariance, the construction yields a state, given by equation (3.15), that is a Gaussian-weighted superposition of the time-translated states $\exp(-i\hat{H}t)|\phi \rangle$. This state represents the galaxy existing in time only for a duration of the order of *σ*_*t*_ and is analogous to equation (2.1) which represents the centre of mass of the galaxy existing only in a spatial region with a size of the order of *σ*_*x*_. Our construction, therefore, allows for the same kind of quantum state in time as in space, in the absence of T violation. In other words, there is a symmetry between time and space for quantum states in this special case. As discussed in the Introduction, this symmetry comes at the cost of the non-conservation of mass.

### (c) Violation of time-reversal invariance

Next, we examine the quite different situation of T violation where λ≠0 and ${\hat{H}}_{F}\neq {\hat{H}}_{B}$. In that case, the amplitudes for different virtual paths to the same point in time, as illustrated in figure 2*b*, can interfere leading to undulations in *I*_*N*−*n*,*n*_(*z*) as a function of *n*. To find the values of *n* where the modulus of the interference function *I*_*N*−*n*,*n*_(*z*) is maximized it is sufficient to look for the position, where |*I*_*N*−*n*,*n*_(*z*)| is unchanged for consecutive values of *n*, i.e. where |*I*_*N*−(*n*−1),*n*−1_(*z*)|=|*I*_*N*−*n*,*n*_(*z*)|. This condition reduces, on using equation (3.10) and performing some algebraic manipulation, to |sin⁡[(N+1−n)z/2]|=|sin⁡(nz/2)|. Note that equations (3.9) and (3.10) imply *z*=*δt*^2^λ and given $\delta t=\sigma_{t}/\sqrt{N}$ from equation (3.4), this means *z* is inversely proportional to *N*; thus we let *z*=*θ*/*N*, where

$$\theta =\sigma_{t}^{2}\lambda$$

is the coefficient of proportionality (i.e. *θ* is independent of *N*). Hence we wish to know the values of *n* that satisfy |sin⁡[(N+1−n)θ/2N]|=|sin⁡(nθ/2N)|. Writing *x*=*θ*(*N*+1)/2*N* and *y*=*nθ*/2*N* transforms this equation into |sin⁡(x−y)|=|sin⁡(y)| which has the solutions *y*=(*x*−*π*)/2+*mπ* for integer *m*. Re-expressing the solutions in terms of *n* then gives

$$n=\frac{N+1}{2}+\frac{N(2m-1)\pi }{\theta }.$$

The modulus of the interference function reaches a maximum value at this value of *n* and one less (i.e. for *n*−1). Taking the midpoint and choosing the particular values *m*=0,1 then gives the positions of two maxima (or ‘peaks’) at *n*=*n*_±_ where

3.16 $$n_{\pm }=N\left(\frac{1}{2}\pm \frac{\pi }{\theta }\right).$$

Substituting *n*_±_ for *n* in equation (3.12) gives the corresponding clock times as

3.17 $$\pm t_{c}^{\left(\mathrm{peak}\right)}=(2n_{\pm }-N)\delta t=\pm \frac{2\pi \sigma_{t}\sqrt{N}}{\theta },$$

where $t_{c}^{\left(\mathrm{peak}\right)}$ is defined to be positive.

The modulus of the interference function equation (3.10) is shown in Section A of the electronic supplementary material to be approximately Gaussian about these maxima, which allows us to write |*Υ*_λ_〉_*N*_ in equation (3.9) as a superposition of two states as follows

3.18 $${\left|\Upsilon_{\lambda }\right\rangle }_{N}\propto {\left|\Upsilon_{\lambda }^{\left(+\right)}\right\rangle }_{N}+{\left|\Upsilon_{\lambda }^{\left(-\right)}\right\rangle }_{N},$$

where

3.19 $${\left|\Upsilon_{\lambda }^{\left(\pm \right)}\right\rangle }_{N}\propto \sum_{n=0}^{N}f_{n}^{\left(\pm \right)}g_{n}^{\left(\pm \right)}\exp[i{\hat{H}}_{B}(N-n)\delta t]\exp[-i{\hat{H}}_{F}n\delta t]|\phi \rangle$$

for 2*π*<*θ*<4*π*. Here

3.20 $$f_{n}^{\left(\pm \right)}=\exp\left\{-\frac{i[n_{+}n_{-}-{\left(n-n_{\pm }\right)}^{2}]\theta }{2N}\right\}$$

and

3.21 $$g_{n}^{\left(\pm \right)}=\exp\left[-\frac{{\left(n-n_{\pm }\right)}^{2}|\theta \tan(\theta /4)|}{2N}\right]$$

are a complex phase function and a Gaussian weighting function, respectively. Keeping in mind the definition of the clock time *t*_c_ from equation (3.12) for the state in equation (3.11), we find that ${\left|\Upsilon_{\lambda }^{\left(\pm \right)}\right\rangle }_{N}$ is a Gaussian-weighted superposition of states over a range of clock times with a mean of $t_{c}=\pm t_{c}^{\left(\mathrm{peak}\right)}$ and a variance of ${\left(\Delta t_{c}\right)}^{2}\approx 2/|\lambda \tan(\theta /4)|$. In other words, the states ${\left|\Upsilon_{\lambda }^{\left(+\right)}\right\rangle }_{N}$ and ${\left|\Upsilon_{\lambda }^{\left(-\right)}\right\rangle }_{N}$ represent the universe localized in time for a duration of the order of Δ*t*_c_ about the mean times $t_{c}=t_{c}^{\left(\mathrm{peak}\right)}$ and $t_{c}=-t_{c}^{\left(\mathrm{peak}\right)}$, respectively.

The symmetry of the clock times associated with ${\left|\Upsilon_{\lambda }^{\left(+\right)}\right\rangle }_{N}$ and ${\left|\Upsilon_{\lambda }^{\left(-\right)}\right\rangle }_{N}$ about the time *t*_c_=0 reflects the symmetry of the construction equations (3.1) and (3.2) which has no bias towards one direction of time or the other. Moreover, if the state |*ϕ*〉 is T-invariant (i.e. if $\hat{T}|\phi \rangle \propto |\phi \rangle$) and we shall assume that it is, then $\hat{T}{\left|\Upsilon_{\lambda }^{\left(+\right)}\right\rangle }_{N}\propto {\left|\Upsilon_{\lambda }^{\left(-\right)}\right\rangle }_{N}$ and $\hat{T}{\left|\Upsilon_{\lambda }\right\rangle }_{N}\propto {\left|\Upsilon_{\lambda }\right\rangle }_{N}$. This symmetry also arises in time-symmetric cosmological and gravitational studies of the direction of time [51],[52]. As the time evolution in one component of the superposition in equation (3.18) is mirrored in the other, it suffices for us to consider just ${\left|\Upsilon_{\lambda }^{\left(+\right)}\right\rangle }_{N}$ and its corresponding value of $t_{c}^{\left(\mathrm{peak}\right)}=2\pi \sqrt{N}\sigma_{t}/\theta$. Accordingly, we will call this value of $t_{c}^{\left(\mathrm{peak}\right)}$ the *representative clock time* and use it to label the whole state |*Υ*_λ_〉_*N*_. The minimum representative clock time of a state in the set ***υ***_λ_ is found, using equation (3.17) with $N=N_{\min}^{\left(\mathrm{time}\right)}=\sigma_{t}^{2}/\delta t_{\min}^{2}$ and $\theta =\sigma_{t}^{2}\lambda$, to be

3.22 $$t_{c,\min}^{\left(\mathrm{peak}\right)}=\frac{2\pi }{\lambda \delta t_{\min}}.$$

A discussion of the values of λ and *δt*_min_ in relation to *t*^(peak)^_*c*,min_ is given in Section C of the electronic supplementary material.

**Figure 4.**
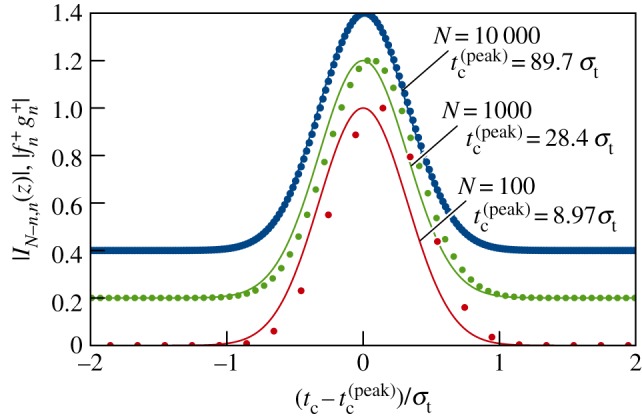
|*I*_*N*−*n*,*n*_(*z*)| plotted as a function of the scaled clock time $(t_{c}-t_{c}^{\left(\mathrm{peak}\right)})/\sigma_{t}$, where *t*_c_=(2*n*−*N*)*δt*. The points $\left(|I_{N-n,n}(z)|,(t_{c}-t_{c}^{\left(\mathrm{peak}\right)})/\sigma_{t}\right)$ are generated parametrically by varying *n*. The dots represent the exact values from equation (3.10), and the solid curves represent the approximation given by $\left|f_{n}^{+}g_{n}^{+}\right|$ in equation (3.19). The numerical values used are *z*=*θ*/*N*, where *θ*=2.23*π* and *N*=100 (red), *N*=1000 (green) and *N*=10 000 (blue). For clarity, the functions have been scaled to give a maximum of unity, and the green (*N*=1000) and blue (*N*=10 000) dots and curves have been displaced vertically by 0.2 and 0.4, respectively. Note that the value of *t*^(peak)^_*c*_ varies as $\sqrt{N}$.

Figure 4 compares the coefficients *I*_*N*−*n*,*n*_(*z*) of the state |*Υ*_λ_〉_*N*_ in equation (3.9) with their Gaussian approximation $f_{n}^{+}g_{n}^{+}$ in equation (3.19) near a maximum. The coefficients have been plotted as a function of $(t_{c}-t_{c}^{\left(\mathrm{peak}\right)})/\sigma_{t}$ to centre them in the figure, where *t*^(peak)^_*c*_ is the position of the maxima given by equation (3.17). As in figure 3, the values of *N* have been chosen purposely to exaggerate the discreteness of the state |*Υ*_λ_〉_*N*_. However, for every |*Υ*_λ_〉_*N*_∈***υ***_λ_ in equation (3.1), the values of *N* are sufficiently large (i.e. $N\geq N_{\min}^{\left(\mathrm{time}\right)}$) that the locus of points representing *I*_*N*−*n*,*n*_(*z*) is essentially equivalent to the Gaussian approximation $f_{n}^{+}g_{n}^{+}$ up to a proportionality constant. It follows that each |*Υ*_λ_〉_*N*_∈***υ***_λ_ is physically indistinguishable from a state given by equations (3.18) and (3.19) with the same value of *N* but where the sum over *n* in equation (3.19) is replaced with its integral equivalent.

**Figure 5.**
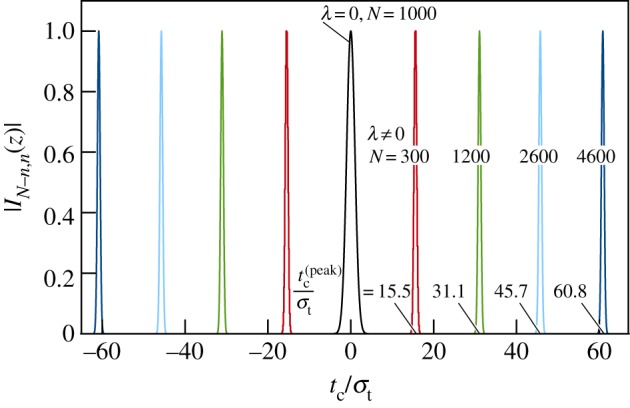
|*I*_*N*−*n*,*n*_(*z*)| as a function of the scaled clock time *t*_c_/*σ*_*t*_, where *t*_c_= (2*n*−*N*)*δt* for different values of λ and *N*. As in figure 4, the points (|*I*_*N*−*n*,*n*_(*z*)|,*t*_c_/*σ*_*t*_) are generated parametrically by varying *n*. For clarity, in each case, straight lines connect consecutive discrete points of |*I*_*N*−*n*,*n*_(*z*)| to form a continuous curve. However, as discussed in the text and illustrated by figure 4, the locus of points representing |*I*_*N*−*n*,*n*_(*z*)| is essentially a continuous curve for *N*≥*N*^(time)^_min_. The black curve represents the T-invariant case (i.e. λ=0) and has been generated for *N*=1000. It does not visibly change with increasing values of *N*. The remaining curves represent the T violation case (i.e. λ≠0) for *θ*=2.23*π* and a range of *N* values as follows: red curve for *N*=300 and *t*^(peak)^_*c*_=15.5 *σ*_*t*_, green curve for *N*=1200 and *t*^(peak)^_*c*_=31.1 *σ*_*t*_, light blue curve for *N*=2600 and *t*^(peak)^_*c*_=45.7 *σ*_*t*_, and dark blue curve for *N*=4600 and *t*^(peak)^_*c*_= 60.8 *σ*_*t*_. All curves have been scaled to give a maximum of unity.

The broad properties of the states |*Υ*_λ_〉_*N*_ are illustrated in figure 5 which shows |*I*_*N*−*n*,*n*_(*z*)| plotted as a function of the scaled clock time *t*_c_/*σ*_*t*_. The black curve corresponds to the time-reversal invariance case where λ=0 (and so $\theta =\sigma_{t}^{2}\lambda =0$). All other curves correspond to the violation of time-reversal invariance (i.e. λ≠0) and have been generated for *θ*=2.23*π* which gives the minimum uncertainty in energy and time (see Section B of the electronic supplementary material for details). The figure illustrates how the location of the maxima at $t_{c}=\pm t_{c}^{\left(\mathrm{peak}\right)}$ increases with *N* as given by equation (3.17).

For clarity, |*I*_*N*−*n*,*n*_(*z*)| is plotted in figure 5 only for a select few values of *N* for which the peaks in the corresponding curves are widely separated. To see how close the peaks can be, consider the difference *δt*^(peak)^_*c*_ in the representative clock times *t*^(peak)^_*c*_ of states |*Υ*_λ_〉_*N*_ with consecutive values of *N*, which is found from equation (3.17) to be

$$\delta t_{c}^{\left(\mathrm{peak}\right)}=\frac{2\pi \sigma_{t}\sqrt{N+1}}{\theta }-\frac{2\pi \sigma_{t}\sqrt{N}}{\theta }\approx \frac{\sigma_{t}\pi }{\theta \sqrt{N}}$$

for large *N*. Noting that $N\geq N_{\min}^{\left(\mathrm{time}\right)}=\sigma_{t}^{2}/\delta t_{\min}^{2}$ gives *δt*^(peak)^_*c*_≤(*π*/*θ*)*δt*_min_ and as 2*π*<*θ*<4*π* we find

$$\delta t_{c}^{\left(\mathrm{peak}\right)}<\frac{1}{2}\delta t_{\min}.$$

Hence, for any given time $t>t_{c,\min}^{\left(\mathrm{peak}\right)}$, there is a state in the set ***υ***_λ_ given by equation (3.1) whose representative clock time *t*^(peak)^_*c*_ is equal to *t* to within the resolution limit *δt*_min_.

Figure 5 clearly shows that the inclusion of the violation of time-reversal invariance *dramatically* changes the set ***υ***_λ_ in equation (3.1) from one containing elements that are physically equivalent (represented by the black curve), to one containing states that are diverging in time (other curves). This striking outcome warrants careful consideration. Both sets ***υ***_λ=0_ and ***υ***_λ≠0_ have the same mathematical construction given by equation (3.1); the difference between them is due solely to the phenomenological Hamiltonian and whether it respects T symmetry (λ=0) or not (λ≠0). All the states in ***υ***_λ=0_ are physically equivalent to a unique state, |*Υ*_0_〉, which represents the galaxy as existing for one particular finite period in time. This constitutes phenomenology associated with T symmetry. In contrast, with T violation there are infinitely many different states in the set ***υ***_λ≠0_. There is no reason to suppose that any of them has special significance and so, by default, all states in ***υ***_λ≠0_ have equal status in representing the state of the galaxy in time. This *pluralism* constitutes phenomenology associated with T violation. That different states can equally represent the galaxy is not contradictory, because each state represents the galaxy at a different representative clock time. In fact, the same pluralism is assumed in conventional quantum physics, and is the root of the asymmetry between time and space discussed in the Introduction.

### (d) Impact for quantum states in time and space

These remarkable results manifest a fundamental difference between quantum states in time and space. All the states in the set ***ψ***, irrespective of whether the discrete symmetries are obeyed or not, represent the galaxy existing only in a region of order *σ*_*x*_ near *x*=0. Likewise, all the states in the set ***υ***_λ=0_ associated with T symmetry represent the galaxy existing only for a duration of order *σ*_*t*_ near *t*_c_=0. The fact that the states in the set ***υ***_λ=0_ do not conserve mass is testament to mass conservation not being an explicit property of the construction defined by equations (3.1) and (3.2). But for a set ***υ***_λ′_ associated with T violation with λ′≠0, for any given time *t*≥*t*^(peak)^_*c*,min_, we have just seen that there is a state |*Υ*_λ′_〉_*N*_∈***υ***_λ′_, whose representative clock time *t*^(peak)^_*c*_ is equal to *t* to within the resolution limit *δt*_min_. In other words, the set ***υ***_λ′_ contains a state that represents the galaxy's existence at each corresponding moment in time. That being the case, it would not be unreasonable to regard the set as representing a *history* of the universe. It follows that the set ***υ***_λ′_ represents the persistence of the mass of the galaxy over the same period of time, in so far as the Hamiltonians ${\hat{H}}_{F}$ and ${\hat{H}}_{B}$ conserve mass. This raises a subtle point regarding conservation laws; while they may be due to deep principles (such as Noether's theorem), they are not manifested in quantum mechanics unless the state persists over a period of time. The crucial point being that in conventional quantum mechanics, the persistence of the state is essentially *axiomatic* and ensured by adopting a compliant dynamical equation of motion whereas here it arises *phenomenologically* as a property of the set of states ***υ***_λ′_. Finally, on comparing the two sets ***υ***_λ=0_ and ***υ***_λ′≠0_, one could even venture to say that T violation, in effect, *causes* the contents of the universe to be translated or, indeed, to *evolve*, over an unbounded period of time.

## 4. Emergence of conventional quantum mechanics

### (a) Coarse graining over time

The spread of the state |*Υ*_λ_〉_*N*_ along the time axis, as illustrated by the plots of |*I*_*N*−*n*,*n*_(*z*)| in figure 5, represents a significant departure from conventional quantum mechanics for which states are interpreted as having no extension in time. Nevertheless, the conventional formalism can be recovered in the following way. Imagine that observations of the galaxy are made with a resolution in time that is much larger than the width of the Gaussian weighting function $g_{n}^{\pm }$ in equation (3.19). Under such coarse graining, the summation in equation (3.19) can be replaced by the term corresponding to the maximum in $g_{n}^{\pm }$ and so, for example,

$${\left|\Upsilon_{\lambda }^{\left(+\right)}\right\rangle }_{N}\underset{∼}{\propto }\exp[i{\hat{H}}_{B}(N-n_{+})\delta t]\exp(-i{\hat{H}}_{F}n_{+}\delta t)|\phi \rangle .$$

We can re-express this state in terms of its representative clock time, $t_{c}^{\left(\mathrm{peak}\right)}$, which we shall shorten to *t*_c_ for brevity, as

4.1 $${\left|\Upsilon_{\lambda }^{\left(+\right)}\right\rangle }_{N}\underset{∼}{\propto }\exp(i{\hat{H}}_{B}t_{c}a_{-})\exp(-i{\hat{H}}_{F}t_{c}a_{+})|\phi \rangle ,$$

where *a*_±_=*n*_±_/(*n*_+_−*n*_−_) and we have used *t*_c_=(2*n*_+_−*N*)*δt*=(*n*_+_−*n*_−_)*δt* and *N*−*n*_+_=*n*_−_. At this level of coarse graining, the time step *δt* is effectively zero, and *t*_c_ is effectively a continuous variable. Making use of the Baker–Campbell–Hausdorff formula [50] in equation (4.1) yields

4.2 $${\left|\Upsilon_{\lambda }^{\left(+\right)}\right\rangle }_{N}\underset{∼}{\propto }\exp\left(\frac{1}{2}ia_{+}a_{-}t_{c}^{2}\lambda \right)\exp\left[-i\left({\hat{H}}_{F}a_{+}-{\hat{H}}_{B}a_{-}\right)t_{c}\right]|\phi \rangle .$$

The complex phase factor can be accommodated by transforming to a new state, $\left|\tilde{\Upsilon }(t_{c})\right\rangle$, as follows

4.3 $$|\tilde{\Upsilon }(t_{c})\rangle =\exp\left(-\frac{1}{2}ia_{+}a_{-}t_{c}^{2}\lambda \right){\left|\Upsilon_{\lambda }^{\left(+\right)}\right\rangle }_{N}\propto \exp\left[-i\left({\hat{H}}_{F}a_{+}-{\hat{H}}_{B}a_{-}\right)t_{c}\right]|\phi \rangle .$$

On taking the derivative with respect to *t*_c,_ we recover Schrödinger's equation,

4.4 $$\frac{d}{dt_{c}}|\tilde{\Upsilon }(t_{c})\rangle \underset{∼}{\propto }-i\left({\hat{H}}_{F}a_{+}-{\hat{H}}_{B}a_{-}\right)|\tilde{\Upsilon }(t_{c})\rangle .$$

Here, the coarse-grained Hamiltonian $\left({\hat{H}}_{F}a_{+}-{\hat{H}}_{B}a_{-}\right)$ is a linear combination of ${\hat{H}}_{F}$ and ${\hat{H}}_{B}$ due to the fact that the quantum virtual path involves contributions from both.

Note that the differential equation (4.4) does not depend on the state |*ϕ*〉. The ambiguity associated with loosely specifying |*ϕ*〉 as being sharply defined in time does not play a role here. It is true that different choices for |*ϕ*〉 will lead to different states $\left|\tilde{\Upsilon }(t_{c})\right\rangle$, but that is no concern when the goal is to show that conventional quantum mechanics is recovered. The fact that equation (4.4) results for all allowed choices of |*ϕ*〉 is all that is needed for this.

It is useful at this point to divide the galaxy into two non-interacting subsystems, one whose Hamiltonian ${\hat{H}}^{\left(i\right)}={\hat{T}}^{-1}{\hat{H}}^{\left(i\right)}\hat{T}$ is T-invariant and the remainder whose Hamiltonian ${\hat{H}}_{F}^{\left(v\right)}={\hat{T}}^{-1}{\hat{H}}_{B}^{\left(v\right)}\hat{T}\neq {\hat{H}}_{B}^{\left(v\right)}$ is T-violating; in that case, we can write

4.5 $${\hat{H}}_{F}={\hat{H}}^{\left(i\right)}⊗{\hat{1}}^{\left(v\right)}+{\hat{1}}^{\left(i\right)}⊗{\hat{H}}_{F}^{\left(v\right)}\;\mathrm{and}\;{\hat{H}}_{B}={\hat{H}}^{\left(i\right)}⊗{\hat{1}}^{\left(v\right)}+{\hat{1}}^{\left(i\right)}⊗{\hat{H}}_{B}^{\left(v\right)},$$

where the superscripts ‘i’ and ‘v’ label operators associated with the state space of the T-invariant and T-violating Hamiltonians, respectively, and ${\hat{1}}^{(\cdot )}$ is an appropriate identity operator. Equation (4.4) can then be rewritten as

4.6 $$\frac{d}{dt_{c}}|\tilde{\Upsilon }(t_{c})\rangle \underset{∼}{\propto }-i\left({\hat{H}}^{\left(i\right)}⊗{\hat{1}}^{\left(v\right)}+{\hat{1}}^{\left(i\right)}⊗{\hat{H}}_{\mathrm{phen}}^{\left(v\right)}\right)|\tilde{\Upsilon }(t_{c})\rangle ,$$

where ${\hat{H}}_{\mathrm{phen}}^{\left(v\right)}={\hat{H}}_{F}^{\left(v\right)}a_{+}-{\hat{H}}_{B}^{\left(v\right)}a_{-}$ is the phenomenological Hamiltonian for the T-violating subsystem.

It is straightforward to show that the commutator of ${\hat{H}}_{\mathrm{phen}}^{\left(v\right)}={\hat{H}}_{F}^{\left(v\right)}a_{+}-{\hat{H}}_{B}^{\left(v\right)}a_{-}$ with its time-reversed version is

$$\left[{\hat{H}}_{\mathrm{phen}}^{\left(v\right)},\;{\hat{T}}^{-1}{\hat{H}}_{\mathrm{phen}}^{\left(v\right)}\hat{T}\right]=-i\frac{\theta }{2\pi }\lambda$$

which is *θ*/2*π* times the commutator $\left[{\hat{H}}_{F}^{\left(v\right)},{\hat{H}}_{B}^{\left(v\right)}\right]$. Thus, in principle, the commutation relation could be used to distinguish the phenomenological Hamiltonians ${\hat{H}}_{\mathrm{phen}}^{\left(v\right)}$ and ${\hat{T}}^{-1}{\hat{H}}_{\mathrm{phen}}^{\left(v\right)}\hat{T}$ from the more elementary versions ${\hat{H}}_{F}^{\left(v\right)}$ and ${\hat{H}}_{B}^{\left(v\right)}$.

### (b) Conventional formalism and potential experimental tests

These results are important because they not only show how the conventional formalism of quantum mechanics is recovered, but they also show how the construction introduced here may be verified experimentally. To see this consider the following three points. First, equation (4.6) shows that the T-invariant subsystem behaves in accord with the conventional Hamiltonian ${\hat{H}}^{\left(i\right)}$ with respect to clock time *t*_c_. This means that conventional quantum mechanics is recovered for this subsystem. Second, equation (4.6) shows that, owing to the coarse graining, the role of the clock time *t*_c_ has been reduced from being a physical variable that describes the location and uncertainty of the galaxy with respect to time as illustrated in figure 5, to being simply a parameter that labels a different state in the set ***υ***_λ_ according to the time $t_{c}=t_{c}^{\left(\mathrm{peak}\right)}$ of the maximum in $g_{n}^{+}$. Indeed, its demoted role is the very reason we are able to recover Schrödinger's equation. Third, any experiments involving T-violating matter that are performed by observers in the galaxy would give results that are consistent with equation (4.6) and so they would provide evidence of the phenomenological Hamiltonian ${\hat{H}}_{\mathrm{phen}}^{\left(v\right)}$ in the same way that experiments in our universe give evidence of the Hamiltonian associated with meson decay. Any demonstration that ${\hat{H}}_{\mathrm{phen}}^{\left(v\right)}$ differs from the more elemental Hamiltonians ${\hat{H}}_{F}^{\left(v\right)}$ and ${\hat{H}}_{B}^{\left(v\right)}$ represents a ‘smoking gun’ for the construction introduced here. Of course, this specific result cannot be used in practice because it applies to the simple model of T violation chosen here for its clarity rather than accuracy, and also because the present knowledge of T violating Hamiltonians is based on empirical results and so it is limited to the phenomenological version of the Hamiltonians. More realistic models of the universe and T violating mechanisms may provide experimentally testable predictions, such as novel deviations from exponential decay for T violating matter or local variations in clock time. But these are beyond the scope of the present work whose aim is to show, in the clearest way possible, how T violation may underlie differences between time and space.

## 5. Discussion

We began by drawing attention to the asymmetry between time and space in conventional quantum theory where states are presumed to undergo continuous translation over time, whereas there is no corresponding presumption about states necessarily undergoing translations over space. We set out to explore an alternate possibility by introducing a new quantum formalism that gives both space and time analogous quantum descriptions. In developing the formalism, we paid particular attention to subtle mathematical details that play no significant role in conventional quantum mechanics. These details involve explicitly taking into account the P and T symmetry operations, translations of states in space and time, and fundamental limits of precision. We incorporated them in a mathematical construction where quantum states are represented as a superposition of random paths in space or time. We found that with no P or T symmetry violations, quantum states had analogous representations in space and time: just as matter can be represented as existing only in a finite region of space, it can also be represented as existing only for a finite interval of time. Clearly, the price we pay for this symmetry is absence of the conservation of mass. However, with the violation of T symmetry, dramatic differences between the representation of quantum states in space and time arise through the quantum interference between different paths. The state (and the matter it describes) is found to persist over an unbounded range of time values. This result gives a new appreciation of conservation laws: while they may be due to deep principles, they are not manifested unless the state persists over a sufficient period of time. The Schrödinger equation of conventional quantum mechanics, where time is reduced to a classical parameter, also emerges as a result of coarse graining over time. As such, T violation is seen in the new formalism as being responsible for fundamental differences between space and time in conventional quantum mechanics.

The new formalism may also help resolve other perplexing issues associated with space and time. For example, the arrows of time indicate a preferred direction from ‘past’ to ‘future’ [53], but there is no analogous preferred direction of space. The new formalism appears to offer a basis for understanding why. Indeed, the set of states in time, ***υ***_λ_ for λ≠0 in equation (3.1), has a natural order over time in the following sense. First recall that our interpretation of equation (3.3) is that $\exp(-i{\hat{H}}_{F}t)$ and $\exp(i{\hat{H}}_{B}t)$ are associated with physical evolution in different directions of time, whereas the inverses $\exp(i{\hat{H}}_{F}t)$ and $\exp(-i{\hat{H}}_{B}t)$ are associated with the mathematical operations of rewinding that physical evolution. Within this context, the coarse-grained state $\left|\tilde{\Upsilon }(t_{c})\right\rangle$ in equation (4.3) is interpreted as resulting from evolution by *t*_c_*a*_+_ in the positive direction of time and *t*_c_*a*_−_ in the reverse direction, giving a net evolution of *t*_c_(*a*_+_−*a*_−_)=*t*_c_ in time from the state |*ϕ*〉. Correspondingly, the state $\left|\tilde{\Upsilon }(t_{c}^{\prime })\right\rangle$ with *t*′_c_>*t*_c_ represents a *more evolved state* than $\left|\tilde{\Upsilon }(t_{c})\right\rangle$. In fact writing

5.1 $$|\tilde{\Upsilon }(t_{c}^{\prime })\rangle \propto \exp[-i({\hat{H}}_{F}a_{+}-{\hat{H}}_{B}a_{-})\Delta t]|\tilde{\Upsilon }(t_{c})\rangle ,$$

where Δ*t*=*t*′_c_−*t*_c_>0 shows that $\left|\tilde{\Upsilon }(t_{c}^{\prime })\right\rangle$ evolves from $\left|\tilde{\Upsilon }(t_{c})\right\rangle$. One might be tempted to argue that we could equally well regard $\left|\tilde{\Upsilon }(t_{c})\right\rangle$ as evolving from $|\tilde{\Upsilon }(t_{c}^{\prime })\rangle ,$ because

5.2 $$|\tilde{\Upsilon }(t_{c})\rangle \propto \exp[i({\hat{H}}_{F}a_{+}-{\hat{H}}_{B}a_{-})\Delta t]|\tilde{\Upsilon }(t_{c}^{\prime })\rangle ,$$

but doing so would be inconsistent with our interpretation of equation (3.3). According to that interpretation, equation (5.2) represents the mathematical *rewinding* of the physical evolution represented by equation (5.1). Note that the state $\left|\tilde{\Upsilon }(t_{c})\right\rangle$ is a coarse-grained version of the component ${\left|\Upsilon_{\lambda }^{\left(+\right)}\right\rangle }_{N}$ of |*Υ*_λ_〉_*N*_ in equation (3.18); an analogous argument also applies to the coarse-grained version of the other component ${\left|\Upsilon_{\lambda }^{\left(-\right)}\right\rangle }_{N}$, and thus to the whole state |*Υ*_λ_〉_*N*_. Hence, the set of states ***υ***_λ_ for λ≠0 are ordered by the degree of time evolution from the state |*ϕ*〉. This gives two preferred directions of time away from the origin of the time axis and so represents a *symmetric arrow of time*. Time-symmetric arrows have also been explored by Carroll *et al.* and Barbour *et al.* [51],[52]. In stark contrast, there is no analogous ordering for ***ψ*** in equation (2.6), the set of states distributed over space. Indeed, all the states in ***ψ*** are physically indistinguishable. Also the ordering of the set ***υ***_λ_ vanishes at λ=0 which corresponds to T symmetry. It appears, therefore, that T violation is also responsible giving time a direction (in the sense of orientating time away from the occurrence of |*ϕ*〉).

In addition to these conceptual results, the new formalism was also found to have potential experimentally testable consequences. Indeed, for a subsystem associated with T violation, the formalism predicts that the experimentally determined Hamiltonian, ${\hat{H}}_{\mathrm{phen}}^{\left(v\right)}$ in equation (4.6), will be different to the Hamiltonians, ${\hat{H}}_{F}^{\left(v\right)}$ or ${\hat{H}}_{B}^{\left(v\right)}$ in equation (4.5), associated with conventional quantum mechanics. Further work is needed to develop feasible experiments for testing predicted departures from conventional theory like this. An experimental verification of the new formalism would have profound impact on our understanding of time.

In conclusion, the importance of Feynman's sums over paths for describing quantum phenomena is well beyond doubt [43]. A distinctive feature of the quantum virtual paths in the new formalism is that they explicitly take into account the violation of T symmetry. The new formalism has the advantage of giving *time and space an equal footing at a fundamental level* while allowing familiar differences, such as matter being localized in space but undergoing unbounded evolution in time, to arise *phenomenologically* due to the fact that T violation is a property of translations in time and not space. As such, the violation of the discrete symmetries is seen to play a defining role in the quantum nature of time and space.

## Supplementary Material

Supplementary Material for “Quantum asymmetry between time and space”

## Data accessibility

Electronic supplementary material is available at http://dx.doi.org/10.1098/rspa.2015.0670 or via http://rspa.royalsocietypublishing.org.

## References

[1] Lee TD, Yang CN 1956 Question of parity conservation in weak interactions. *Phys. Rev.* 104, 254–258. (doi:10.1103/PhysRev.104.254)

[2] Christenson JH, Cronin JW, Fitch VL, Turlay R 1964 Evidence for the 2*π* decay of the *K*_2_^0^ meson. *Phys. Rev. Lett.* 13, 138–140. (doi:10.1103/PhysRevLett.13.138)

[3] Angelopoulos A *et al.* 1998 First direct observation of time-reversal non-invariance in the neutral-kaon system. *Phys. Lett. B* 444, 43–51. (doi:10.1016/S0370-2693(98)01356-2)

[4] Sehgal LM, van Leusen J 1999 Violation of time reversal invariance in the decays *K*_*L*_→*π*^+^*π*^−^*γ* and *K*_*L*_→*π*^+^*π*^−^*e*^+^*e*^−^. *Phys. Rev. Lett.* 83, 4933–4936. (doi:10.1103/PhysRevLett.83.4933)

[5] Alvarez-Gaume L, Kounnas C, Lola S, Pavlopoulos P 1999 Violation of time-reversal invariance and CPLEAR measurements. *Phys. Lett. A* 458, 347–354. (doi:10.1016/S0370-2693(99)00520-1)

[6] Lees JP *et al.* 2012 Observation of time-reversal violation in the *B*^0^ meson system. *Phys. Rev. Lett.* 109, 211801 (doi:10.1103/PhysRevLett.109.211801) 23215586 10.1103/PhysRevLett.109.211801

[7] Cabibbo N 1963 Unitary symmetry and leptonic decays. *Phys. Rev. Lett.* 10, 531–533. (doi:10.1103/PhysRevLett.10.531)

[8] Kobayashi M, Maskawa T 1973 CP-violation in the renormalizable theory of weak interaction. *Prog. Theor. Phys.* 49, 652–657. (doi:10.1143/PTP.49.652)

[9] Sakharov AD 1967 Violation of CP symmetry, C asymmetry and baryon asymmetry of the universe. *JETP Lett.* 5, 24–27.

[10] Aharony A 1971 Microscopic irreversibility in the neutral kaon system and the thermodynamical arrow of time I. CPT symmetric case. *Ann. Phys.* 67, 1–18. (doi:10.1016/0003-4916(71)90002-9)

[11] Aharony A 1971 Microscopic irreversibility in the neutral kaon system and the thermodynamical arrow of time II. CPT violating case. *Ann. Phys.* 68, 163–171. (doi:10.1016/0003-4916(71)90245-4)

[12] Gell-Mann M, Hartle JB 1994 Time symmetry and asymmetry in quantum mechanics and quantum cosmology. In *Physical origins of time asymmetry* (eds J Halliwell, J Perez-Mercader, W Zurek), pp. 311–345. Cambridge, UK: Cambridge University Press.

[13] Berger Ch, Sehgal LM 2007 CP violation and arrows of time: evolution of a neutral K or B meson from an incoherent to a coherent state. *Phys. Rev. D* 76, 036003 (doi:10.1103/PhysRevD.76.036003)

[14] Courbage M, Durt T, Saberi Fathi SM 2012 Time decay probability distribution of the neutral meson system and CP-violation. *J. Phys. G, Nucl. Part. Phys.* 39, 045008 (doi:10.1088/0954-3899/39/4/045008)

[15] Datta A, Home D, Raychaudhuri A 1987 A curious Gedanken example of the Einstein–Podolsky–Rosen paradox using CP nonconservation. *Phys. Lett. A* 123, 4–8. (doi:10.1016/0375-9601(87)90749-3)

[16] Finkelstein J, Stapp HP 1987 CP violation does not make faster-then-light communication possible. *Phys. Lett. A* 126, 159–162. (doi:10.1016/0375-9601(87)90451-8)

[17] Hall MJW 1987 Imprecise measurements and non-locality in quantum mechanics. *Phys. Lett. A* 125, 89–91. (doi:10.1016/0375-9601(87)90127-7)

[18] Lindblad G 1987 Comment on a curious gedanken experiment involving superluminal communication. *Phys. Lett. A* 126, 71–72. (doi:10.1016/0375-9601(87)90557-3)

[19] Squires E, Siegwart D 1987 CP violation and the EPR experiment. *Phys. Lett. A* 126, 73–74. (doi:10.1016/0375-9601(87)90558-5)

[20] Datta A, Home D, Raychaudhuri A 1988 Is quantum mechanics with CP nonconservation incompatible with Einstein's locality condition at the statistical level? *Phys. Lett. A* 130, 187–191. (doi:10.1016/0375-9601(88)90590-7)

[21] Corbett JV 1988 Quantum mechanical measurement of non-orthogonal states and a test of non-locality. *Phys. Lett. A* 130, 419–425. (doi:10.1016/0375-9601(88)90701-3)

[22] Clifton RK, Redhead MLG 1988 The compatibility of correlated CP violating systems with statistical locality. *Phys. Lett. A* 126, 295–299. (doi:10.1016/0375-9601(88)90838-9)

[23] Bramon A, Nowakowski M 1999 Bell inequalities for entangled pairs of neutral kaons. *Phys. Rev. Lett.* 83, 1 (doi:10.1103/PhysRevLett.83.1)

[24] Ancochea B, Bramon A, Nowakowski M 1999 Bell inequalities for *K*^0^*K*^0^ pairs from F-resonance decays. *Phys. Rev. D* 60, 094008 (doi:10.1103/PhysRevD.60.094008)

[25] Bertlmann RA, Grimus W, Hiesmayr BC 2001 Bell inequality and CP violation in the neutral kaon system. *Phys. Lett. A* 289, 21–26. (doi:10.1016/S0375-9601(01)00577-1)

[26] Bertlmann RA, Hiesmayr BC 2001 Bell inequalities for entangled kaons and their unitary time evolution. *Phys. Rev. A* 63, 062112 (doi:10.1103/PhysRevA.63.062112)

[27] Andrianov AA, Taron J, Tarrach R 2001 Neutral kaons in medium: decoherence effects. *Phys. Lett. B* 507, 200–206. (doi:10.1016/S0370-2693(01)00463-4)

[28] Barnett SM, Kraemer T 2002 CP violation, EPR correlations and quantum state discrimination. *Phys. Lett. A* 293, 211–215. (doi:10.1016/S0375-9601(02)00012-9)

[29] Samal MK, Home D 2002 Violation of Bell's inequality in neutral kaons system. *Pramana* 59, 289–293. (doi:10.1007/s12043-002-0119-z)

[30] Bertlmann RA, Bramon A, Garbarino G, Hiesmayr BC 2004 Violation of a Bell inequality in particle physics experimentally verified? *Phys. Lett. A* 332, 355–360. (doi:10.1016/j.physleta.2004.10.006)

[31] Genovese M 2004 Entanglement properties of kaons and tests of hidden-variable models. *Phys. Rev. A* 69, 022103 (doi:10.1103/PhysRevA.69.022103)

[32] Courbage M, Durt T, Saberi Fathi SM 2010 Dissipative dynamics of the kaon decay process. *Commun. Nonlinear Sci. Numer. Simulat.* 15, 71–78. (doi:10.1016/j.cnsns.2009.01.020)

[33] Fassarella L 2012 Dispersive quantum systems. *Braz. J. Phys.* 42, 84–99. (doi:10.1007/s13538-011-0053-y)

[34] Bertlmann RA, Durstberger K, Hiesmayr BC 2003 Decoherence of entangled kaons and its connection to entanglement measures. *Phys. Rev. A* 68, 012111 (doi:10.1103/PhysRevA.68.012111)

[35] Bertlmann RA, Hiesmayr BC 2006 Kaonic qubits. *Quantum Inf. Process.* 5, 421–440. (doi:10.1007/s11128-006-0026-1)

[36] Hiesmayr BC, Huber M 2008 Bohr's complementarity relation and the violation of CP symmetry in the neutral kaon system. *Phys. Lett. A* 372, 3608–3615. (doi:10.1016/j.physleta.2008.02.055)

[37] Lu D (2014). http://arxiv.org/abs/1405.6209v1.

[38] Vaccaro JA 2011 T violation and the unidirectionality of time. *Found. Phys.* 41, 1569–1596. (doi:10.1007/s10701-011-9568-x)

[39] Vaccaro JA 2015 T violation and the unidirectionality of time: further details of the interference. *Found. Phys.* 45, 691–706. (doi:10.1007/s10701-015-9896-3)

[40] Wigner EP 1959 *Group theory and its application to the quantum mechanics of atomic spectra*. New York, NY: Academic Press.

[41] Bialynicki-Birula I, Mycielski J 1975 Uncertainty relations for information entropy in wave mechanics. *Commun. Math. Phys.* 44, 129–132. (doi:10.1007/BF01608825)

[42] Venegas-Andraca SE 2012 Quantum walks: a comprehensive review. *Quantum Inf. Process.* 11, 1015–1106. (doi:10.1007/s11128-012-0432-5)

[43] Feynman RP 1948 Space–time approach to non-relativistic quantum mechanics. *Rev. Mod. Phys.* 20, 367–387. (doi:10.1103/RevModPhys.20.367)

[44] Goldstein H, Poole C, Safko J 2002 *Classical mechanics*, 3rd edn San Francisco, CA: Addison-Wesley.

[45] Amelino-Camelia G 1999 Gravity-wave interferometers as quantum-gravity detectors. *Nature* 398, 216–218. (doi:10.1038/18377)

[46] Pegg DT 1991 Time in a quantum mechanical world. *J. Phys. A* 24, 3031–3040. (doi:10.1088/0305-4470/24/13/018)

[47] Moyer CA 2015 Timelines and quantum time operators. *Found. Phys.* 45, 382–403. (doi:10.1007/s10701-015-9870-0)

[48] Ivanov IP, Lavoura L 2013 Geometrical CP violation in the N-Higgs-doublet model. *Eur. Phys. J. C* 73, 2416 (doi:10.1140/epjc/s10052-013-2416-8)

[49] Lee TD 1974 CP nonconservation and spontaneous symmetry breaking. *Phys. Rep.* 9, 143–177. (doi:10.1016/0370-1573(74)90020-9)

[50] Suzuki M 1977 On the convergence of exponential operators—the Zassenhaus formula, BCH formula and systematic approximants. *Commun. Math. Phys.* 57, 193–200. (doi:10.1007/BF01614161)

[51] Carroll SM, Chen J 2004 Spontaneous inflation and the origin of the arrow of time. (http://arxiv.org/abs/hep-th/0410270)

[52] Barbour J, Koslowski T, Mercati F 2014 Identification of a gravitational arrow of time. *Phys. Rev. Lett.* 113, 181101 (doi:10.1103/PhysRevLett.113.181101) 25396357 10.1103/PhysRevLett.113.181101

[53] Price H 1996 *Time's arrow and Archimedes’ point*. New York, NY: Oxford University Press.

