# Supplementary material for: Quantum asymmetry between time and space
Source: Proc Math Phys Eng Sci. 2016 Jan;472(2185):20150670. doi: 10.1098/rspa.2015.0670 (PMC4786044; doi:10.1098/rspa.2015.0670)
Supplement: Supplementary Material for “Quantum asymmetry between time and space” [file rspa20150670supp1.pdf]

SUPPLEMENTARY MATERIAL FOR

Quantum asymmetry between time and space

Joan A. Vaccaro

Centre for Quantum Dynamics, Griffith University, Nathan 4111 Australia

A. Approximate shape of the maxima

An approximate form of the interference function $I_{N-n,n}(z)$ in Eq. (3.10) near its maxima can be found by retaining terms of order $1/\sqrt{N}$ or larger as follows. Substituting $n = n_\pm + k$, where k is an integer, into Eq. (3.10) and using $N - n_\pm = n_\mp$ gives

$$I_{N-n,n}(z) = |I_{N-n_\pm,n_\pm}(z)| \exp[-i(n_\pm + k)(n_\mp - k)z/2] \prod_{r=1}^{k} \frac{\sin[(N+1-r-n_\pm)z/2]}{\sin[(r+n_\pm)z/2]} . \quad \text{(A.1)}$$

Next, substituting for $n_\pm$ and z using Eq. (3.16) and $z = \theta/N$, respectively, using trigonometric identities and performing some algebraic manipulations eventually shows that the iterated product in Eq. (A.1) can be written as

$$\prod_{r=1}^{k} \frac{\sin[(N+1-r-n_\pm)z/2]}{\sin[(r+n_\pm)z/2]} = (-1)^k \prod_{r=1}^{k} \frac{\cos(A)\cos(B) + \sin(A)\sin(B)}{\cos(A)\cos(C) - \sin(A)\sin(C)} \quad \text{(A.2)}$$

with $A = \theta/4$, $B = (r-1)\theta/2N$ and $C = r\theta/2N$. Note that k represents the number of steps in time, each of duration $\delta t = \sigma_t/\sqrt{N}$, from the position of the maximum. To retain only features that are a finite distance in time from the maximum in the limit $N \to \infty$, we need to keep terms where k, and thus r, are of order $\sqrt{N}$. It follows that we can use the approximations $\cos(B) \approx 1$, $\cos(C) \approx 1$, $\sin(B) \approx B$ and $\sin(C) \approx C$ to first order in $1/\sqrt{N}$ in Eq. (A.2), and so

$$\prod_{r=1}^{k} \frac{\sin[(N+1-r-n_\pm)z/2]}{\sin[(r+n_\pm)z/2]} \approx (-1)^k \prod_{r=1}^{k} \frac{1 + B\tan(A)}{1 - C\tan(A)} .$$

As $B \approx C \approx r\theta/2N \ll 1$ to the same order of approximation, we can further approximate this as

$$\prod_{r=1}^{k} \frac{\sin[(N+1-r-n_\pm)z/2]}{\sin[(r+n_\pm)z/2]} \approx (-1)^k \prod_{r=1}^{k} \exp\left[\frac{(2r-1)\theta}{2N} \tan\left(\frac{\theta}{4}\right)\right] ,$$

and then, on evaluating the product on the right side, we eventually find

$$\prod_{r=1}^{k} \frac{\sin[(N+1-r-n_\pm)z/2]}{\sin[(r+n_\pm)z/2]} \approx (-1)^k \exp\left[\frac{k^2\theta}{2N} \tan\left(\frac{\theta}{4}\right)\right] .$$

Substituting into Eq. (A.1) then gives

$$I_{N-n,n}(z) = |I_{N-n_\pm,n_\pm}(z)| \exp\left[-i(n_- n_+ - k^2)\frac{\theta}{2N}\right] \exp\left[\frac{k^2\theta}{2N} \tan\left(\frac{\theta}{4}\right)\right] .$$

The right-most factor is a Gaussian function of k provided $\theta\tan(\theta/4)$ is negative. To ensure that this is the case we set $2\pi < \theta < 4\pi$. It follows from $N - n_+ = n_-$ and the symmetry property $I_{N-m,m}(z) = I_{m,N-m}(z)$ that $I_{N-n_+,n_+}(z) = I_{N-n_-,n_-}(z)$. Thus, noting $k = n - n_\pm$, we find

$$I_{N-n,n}(z) \propto f_n^{(+)} g_n^{(+)} + f_n^{(-)} g_n^{(-)}$$

where $f_n^{(\pm)}$ and $g_n^{(\pm)}$ are defined by Eq. (3.20) and Eq. (3.21), respectively. Substituting this result into Eq. (3.9) then leads to Eq. (3.18).

B. Minimum uncertainty in energy and time

We have defined t_c via Eq. (3.11) and Eq. (3.12) as the time measured by clock devices that are constructed from T-invariant matter to avoid any difficulties that might arise in defining clocks that are constructed from T-violating matter. However, for the particular case here where $[\hat{H}_B, \hat{H}_F] = i\lambda$ there are no such difficulties and a clock constructed from both T-invariant and T-violating matter will consistently register the same clock time t_c irrespective of the path, and the value of t_c will be the same as for a clock that is entirely constructed from T-invariant matter. To see this, consider the two paths represented by $\hat{A}\hat{B}|\phi\rangle$ and $\hat{B}\hat{A}|\phi\rangle$ where $\hat{A} = \exp(-i\hat{H}_F n\delta t)$ and $\hat{B} = \exp[i\hat{H}_B(N-n)\delta t]$. It is straightforward to show using the Baker-Campbell-Hausdorff formula that [1]

$$\hat{A}\hat{B}|\phi\rangle = \exp[-i\lambda n(N-n)\delta t^2]\hat{B}\hat{A}|\phi\rangle \tag{B.3}$$

and so both paths result in the same state apart from a complex phase factor. If we regard the whole universe as being a device that registers clock time t_c and if $|\phi\rangle$ represents $t_c = 0$ then Eq. (B.3) implies that both $\hat{A}\hat{B}|\phi\rangle$ and $\hat{B}\hat{A}|\phi\rangle$ represent it registering the clock time $t_c = (2n - N)\delta t$. The clock time t_c is therefore representative of the whole universe in this case.

Although we do not have an operator corresponding to the clock time t_c, we can still estimate the uncertainty in t_c for the state $|\Upsilon_\lambda^{(\pm)}\rangle_N$ using the following heuristic argument. The sum over n in Eq. (3.19) means that, in addition to any intrinsic uncertainty in the time represented by clocks due to the state $|\phi\rangle$, there is an additional contribution due to the finite width of the Gaussian weighting function $g_n^{(\pm)}$. In fact, taking into account the relationship $t_c = (2n - N)\delta t$, the variance in possible clock time values t_c will be at least $(2\delta t)^2$ times the variance in n due to $|g_n^{(\pm)}|^2$. Thus we can bound the uncertainty in clock time as $(\Delta t_c)^2 \gtrsim 4(\Delta n)^2\delta t^2$ where $(\Delta n)^2 = N/[2|\theta\tan(\theta/4)|]$, and so using $\delta t = \sigma_t/\sqrt{N}$ and $\theta = \sigma_t^2\lambda$ we find

$$(\Delta t_c)^2 \gtrsim \frac{2}{|\lambda\tan(\theta/4)|} \tag{B.4}$$

where $(\Delta t_c)^2 = \overline{t_c^2} - \overline{t_c}^2$ for averages calculated using $|g_n^{(\pm)}|^2$ as the probability distribution.

The variance in Eq. (B.4) depends on the value of θ. Rather than use any value in the allowed range $2\pi < \theta < 4\pi$, it would be useful to have one that has a particular physical meaning. One such value corresponds to minimal uncertainties in t_c, H_F and H_B. The first step in finding it is to use the Robertson-Schrödinger uncertainty relation [2] for the Hamiltonians $\hat{H}_F$ and $\hat{H}_B$:

$$(\Delta H_F)^2(\Delta \hat{H}_B)^2 \ge \tfrac{1}{4}|\langle\{\hat{H}_F, \hat{H}_B\}\rangle - 2\langle\hat{H}_F\rangle\langle\hat{H}_B\rangle|^2 + \tfrac{1}{4}|\langle[\hat{H}_F, \hat{H}_B]\rangle|^2$$

where $\{\hat{A}, \hat{B}\} = \hat{A}\hat{B} + \hat{B}\hat{A}$ and $(\Delta A)^2 = \langle\hat{A}^2\rangle - \langle\hat{A}\rangle^2$ is the variance in $\hat{A}$. As $[\hat{H}_B, \hat{H}_F] = i\lambda$, the minimum of the right side occurs when the covariance is zero:

$$\langle\{\hat{H}_F, \hat{H}_B\}\rangle - 2\langle\hat{H}_F\rangle\langle\hat{H}_B\rangle = 0\,. \tag{B.5}$$

Thus the minimum uncertainty is given by

$$(\Delta H_F)^2(\Delta H_B)^2 = \frac{|\lambda|^2}{4}\,. \tag{B.6}$$

With no bias towards one direction of time or the other, there is correspondingly no bias towards one version of the Hamiltonian or the other and so we take the minimum uncertainty condition for the energy as

$$(\Delta H_F)^2 = (\Delta H_B)^2 = \frac{|\lambda|}{2}\,. \tag{B.7}$$

Next we need to determine the relationship between Δt_c, ΔH_F and ΔH_B. Unfortunately, there has not been any previous study of the time-energy uncertainty relation for the case of T violation where two versions of the Hamiltonian operate, and it would be beyond the scope of this work to analyse it in detail here. Nevertheless, we can glean some insight into the problem as follows.

Consider the operator defined by

$$\hat{H} = \tfrac{1}{2}(\hat{H}_{\rm F} + \hat{H}_{\rm B});$$

it is straightforward to show that $\hat{\mathbf{T}}^{-1}\hat{H}\hat{\mathbf{T}} = \hat{H}$ and so $\hat{H}$ is T invariant. We know from the discussion of Eq. (B.3) that the state $|\psi\rangle = \exp(-i\hat{H}_{\rm F}t_1)\exp[i\hat{H}_{\rm B}t_2]|\phi\rangle$ represents the time $t_{\rm c} = t_1 - t_2$. Rearranging using the Baker-Campbell-Hausdorff formula [1] shows that $|\psi\rangle \propto \exp[-i\hat{H}_{\rm F}(t_1+t_2)]\exp[i\hat{H}2t_2]|\phi\rangle$ and so it follows that the state $\exp[i\hat{H}2t_2]|\phi\rangle$ represents the time $t_{\rm c} = -2t_2$. Thus $\hat{H}$ is clearly a generator of translations in time. A similar argument shows that the operator $\frac{1}{2}(\hat{H}_{\rm F} - \hat{H}_{\rm B})$ does not generate translations in time. This implies that there is a meaningful uncertainty relation for the clock time $t_{\rm c}$ and $\hat{H}$. The uncertainty in $\hat{H}$ is related to that of $H_{\rm F}$ and $H_{\rm B}$ by

$$(\Delta H)^2 = \tfrac{1}{4}\left[(\Delta H_{\rm F})^2 + (\Delta H_{\rm B})^2 + \langle\{\hat{H}_{\rm F}, \hat{H}_{\rm B}\}\rangle - 2\langle\hat{H}_{\rm F}\rangle\langle\hat{H}_{\rm B}\rangle\right] ,$$

and if Eq. (B.7) holds, then so does Eq. (B.5) and we find

$$(\Delta H)^2 = \frac{|\lambda|}{4} . \tag{B.8}$$

It is easy to calculate the product of variances in $t_{\rm c}$ and $\hat{H}$ for a state like Eq. (3.14). In the limit $N\to\infty$ the sum over m in Eq. (3.14) becomes an integral over $t = m\delta t$ and so

$$\lim_{N\to\infty}|\Upsilon_0\rangle_N \propto \int dt\,\exp(-\frac{t^2}{2\sigma_{\rm t}^2})\exp(-i\hat{H}t)|\phi\rangle \tag{B.9}$$

which is the temporal analogy of Eq. (2.2). Replacing $\sigma_{\rm t}^2$ with $2(\Delta t_{\rm c})^2$ and performing the integral in Eq. (B.9) in the eigenbasis of $\hat{H}$ yields

$$\lim_{N\to\infty}|\Upsilon_0\rangle_N \propto \exp[-\hat{H}^2(\Delta t_{\rm c})^2]|\phi\rangle .$$

The state $|\phi\rangle$ is assumed to have a large variance in energy; in the limit that $|\phi\rangle$ is a uniform superposition of the eigenstates of $\hat{H}$, the probability distribution for $\hat{H}$ for the state on the right side becomes a truncated Gaussian [3] with a variance of $(\Delta H)^2 \approx (1-2/\pi)/4(\Delta t_{\rm c})^2$. Hence an approximate energy-time uncertainty relation for this particular class of states is

$$(\Delta H)^2(\Delta t_{\rm c})^2 \approx \frac{1}{4}(1-\frac{2}{\pi}) .$$

We will presume that this result also applies to the states $|\Upsilon_\lambda^{(\pm)}\rangle_N$ in Eq. (3.19) without significant modification. In that case using Eq. (B.8) to replace $(\Delta H)^2$ gives

$$(\Delta t_{\rm c})^2 \approx \frac{1}{|\lambda|}(1-\frac{2}{\pi}) . \tag{B.10}$$

This gives the least uncertainty in clock time for the case where the uncertainty in energy is minimized; to be clear, the uncertainty in $\Delta t_{\rm c}$ can be smaller than that given by Eq. (B.10) provided the uncertainty in energy is higher than the minimum represented by the equality in Eq. (B.6). Comparing Eq. (B.4) and Eq. (B.10) and keeping in mind that $2\pi < \theta < 4\pi$ shows that the minimum uncertainty in energy and time is given by $\tan(\theta/4) \approx -2/(1-2/\pi)$, i.e. for $\theta \approx 2.23\pi$.

Note that the uncertainty $\Delta t_{\rm c}$ is for each of the components $|\Upsilon_\lambda^{(\pm)}\rangle_N$ and not for the whole state $|\Upsilon_\lambda\rangle_N$ in Eq. (3.18). This uncertainty is appropriate from the point of view of an observer within the galaxy for whom the states $|\Upsilon_\lambda^{(\pm)}\rangle_N$ equally describe the state of the universe up to the symmetry given by $\hat{\mathbf{T}}|\Upsilon_\lambda^{(+)}\rangle_N \propto |\Upsilon_\lambda^{(-)}\rangle_N$.

C. Quantifying the T violation

The minimum representative clock time $t_{\rm c,min}^{\rm (peak)}$ for the set Υ_λ defined in Eq. (3.22) and the uncertainty in the clock time $\Delta t_{\rm c}$ defined in Eq. (B.4) are important physical parameters. To estimate their values we need to quantify the minimum physically resolvable time given by $\delta t_{\rm min}$

and the degree of T violation represented by the value of λ. The Planck time, $t_{\rm P} = 5.4 \times 10^{-44}$ s, is widely used as the minimum resolvable time and so we will adopt it here and set $\delta t_{\rm min} = t_{\rm P}$.

Quantifying λ is a rather more difficult. One possibility is to assume that it has of the same order of magnitude as that of meson decay in our universe. The eigenvalue spectrum of the (Hermitian) commutator $i[\hat{H}_{\rm F}, \hat{H}_{\rm B}]$ for meson decay has been estimated to have a Gausian distribution with a mean of zero and a standard deviation of $\sqrt{f} \times 10^{57}\ {\rm s}^{-2}$ where f is the fraction of the estimated 10^{80} particles in the visible universe that contribute to kaon-like T violation [4]. The standard deviation serves as a physically-meaningful value of λ and so we set $\lambda = \sqrt{f} \times 10^{57}\ {\rm s}^{-2}$. Using Eq. (3.22) with these values of λ and $\delta t_{\rm min}$ then gives the minimum representative clock time as

$$t_{\rm c,min}^{\rm (peak)} \approx f^{-1/2} \times 10^{-13}\ {\rm s}\ .$$

Thus Eq. (4.4) and Eq. (4.6) describe the coarse-grained time evolution of the model universe from this time onwards. The corresponding value of the uncertainty in the clock time $\Delta t_{\rm c}$ is, from Eq. (B.10),

$$\Delta t_{\rm c} \approx f^{-1/4} \times 10^{-29}\ {\rm s}\ .$$

Another way to quantify λ is to treat it as if its value is chosen by nature in order that the minimum representative clock time is equal to the minimum time resolution, i.e. to make $t_{\rm c,min}^{\rm (peak)} = \delta t_{\rm min}$. In that case we find, using Eq. (3.22), that

$$\lambda = \frac{2\pi}{\delta t_{\rm min}^2} \tag{C.11}$$

which becomes $\lambda \approx 10^{87}\ {\rm s}^{-2}$ for $\delta t_{\rm min} = t_{\rm P}$. Then using Eq. (B.10) we find the corresponding uncertainty in the clock time is

$$\Delta t_{\rm c} \approx \tfrac{1}{4}\delta t_{\rm min}\ . \tag{C.12}$$

This represents the most extreme situation where Eq. (4.4) and Eq. (4.6) describe the coarse-grained time evolution for all times from $t_{\rm c} = 0$ and the uncertainty in clock time is undetectable.

Finally, we should add that any non-zero value of λ will give rise to the qualitative behaviour described in the main text. However, according to Eq. (3.22), as the value of λ approaches zero, the minimum representative clock time $t_{\rm c,min}^{\rm (peak)}$ becomes correspondingly large and so the desired behaviour is confined to ever larger times $t_{\rm c}^{\rm (peak)}$.

References

1. Suzuki M. 1977 On the convergence of exponential operators—the Zassenhaus formula, BCH formula and systematic approximants. *Commun. Math. Phys.* **57**, 193–200.
2. Robertson HP. 1934 An Indeterminacy relation for several observables and its classical interpretation. *Phys. Rev.* **46**, 794–801.
3. Barr DR, Sherrilla ET. 1999 Mean and variance of truncated normal distributions. *Am. Stat.* **53**, 357–361.
4. Vaccaro JA. 2011 T Violation and the Unidirectionality of Time. *Found. Phys.* **41**, 1569–1596.
